# Lipid metabolism and Type VII secretion systems dominate the genome scale virulence profile of *Mycobacterium tuberculosis* in human dendritic cells

**DOI:** 10.1186/s12864-015-1569-2

**Published:** 2015-05-09

**Authors:** Tom A Mendum, Huihai Wu, Andrzej M Kierzek, Graham R Stewart

**Affiliations:** Department of Microbial and Cellular Sciences, Faculty of Health and Medical Sciences, University of Surrey, Guildford, GU2 7XH UK

**Keywords:** *Mycobacterium tuberculosis*, dendritic cells, transposon library, cholesterol, phenolic glycolipids, sulfolipid, phthiocerol dimycolates, ESX systems, nitrate reductase, PPE genes

## Abstract

**Background:**

*Mycobacterium tuberculosis* continues to kill more people than any other bacterium. Although its archetypal host cell is the macrophage, it also enters, and survives within, dendritic cells (DCs). By modulating the behaviour of the DC, *M. tuberculosis* is able to manipulate the host’s immune response and establish an infection. To identify the *M. tuberculosis* genes required for survival within DCs we infected primary human DCs with an *M. tuberculosis* transposon library and identified mutations with a reduced ability to survive.

**Results:**

Parallel sequencing of the transposon inserts of the surviving mutants identified a large number of genes as being required for optimal intracellular fitness in DCs. Loci whose mutation attenuated intracellular survival included those involved in synthesising cell wall lipids, not only the well-established virulence factors, pDIM and cord factor, but also sulfolipids and PGL, which have not previously been identified as having a direct virulence role in cells. Other attenuated loci included the secretion systems ESX-1, ESX-2 and ESX-4, alongside many PPE genes, implicating a role for ESX-5. In contrast the canonical ESAT-6 family of ESX substrates did not have intra-DC fitness costs suggesting an alternative ESX-1 associated virulence mechanism. With the aid of a gene-nutrient interaction model, metabolic processes such as cholesterol side chain catabolism, nitrate reductase and cysteine-methionine metabolism were also identified as important for survival in DCs.

**Conclusion:**

We conclude that many of the virulence factors required for survival in DC are shared with macrophages, but that survival in DCs also requires several additional functions, such as cysteine-methionine metabolism, PGLs, sulfolipids, ESX systems and PPE genes.

**Electronic supplementary material:**

The online version of this article (doi:10.1186/s12864-015-1569-2) contains supplementary material, which is available to authorized users.

## Introduction

*Mycobacterium tuberculosis* is one of the world’s most successful human pathogens, with almost 10 million new cases every year [1] and more than 2 billion people latently infected. People are usually infected with *M. tuberculosis* by inhaling respired droplets that pass through the upper respiratory tract and deposit the bacilli in the alveoli. Here they are phagocytosed by a range of cells including neutrophils [2,3], macrophages [3] and dendritic cells (DCs) [3,4], all of which contribute to a co-ordinated immunological response to the infection that is often protective. However in some cases *M. tuberculosis* is able to manipulate both the innate, and the subsequent adaptive immune response, to establish an infection that may persist for a lifetime.

Although all these cells engulf *M. tuberculosis* into a membrane bound phagosome, the fundamental functional differences between them result in different phagosomal environments and ultimately different outcomes for each type of phagocyte. For example the neutrophil phagosome remains relatively alkaline and generates a larger oxidative burst [5] than that of macrophages. This is associated with accelerated, necrotic neutrophil death and the signalling of an inflammatory response that is capable of both activating macrophages and causing DCs to mature [6,7].

The primary host of *M. tuberculosis* is often considered to be the unactivated alveolar macrophage. *M. tuberculosis* is able to survive and proliferate in these cells by inhibiting the bactericidal processes of phagosomal fusion [8,9] and acidification [10], and by altering its metabolism to utilise intracellular substrates such as amino acids, fatty acids and cholesterol [11-13]. *M. tuberculosis* also manipulates the cells’ behaviour by modulating immune signalling [14] and antigen presentation [15], and by controlling the timing and mode of cell death [16,17]. Many bacterial genes have been identified as involved in these specific virulence processes [18], both by examining individual mutants, and by using genome-wide genetic screens [19-22].

*M. tuberculosis* are also phagocytosed by immature DCs [23,24], causing them to mature and migrate to the draining lymph nodes where antigens are presented to T cells. *M. tuberculosis* is capable of modulating much of this maturation cascade [25], by for instance lowering MHCI and MHCII presentation to CD8^+^ and CD4^+^ T cells respectively [26], and by altering cytokine profiles [27]. In contrast to macrophages, DCs are thought to be able to limit intracellular *M. tuberculosis* proliferation [28-30], despite *M. tuberculosis* disrupting phagosomal trafficking and preventing phagosomal fusion [31]. Transcriptional studies [29,32] indicate that the DC phagosomal environment has relatively limited connectivity with host cell recycling and biosynthetic pathways and so is nutrient limited, particularly with respect to amino acids and carbon substrates [29]. Other bactericidal stresses such as acidification, and reactive oxygen and nitrogen species that are associated with bactericidal activity in activated macrophages appear to be limited or absent in DCs [29,32,33]. Despite these studies, we still know relatively little about the phagosomal DC environment, and the interactions between it and the bacillus.

In spite of the importance of DCs in the host's response to *M. tuberculosis* infection, there have been no comprehensive studies of the genetic requirements of *M. tuberculosis* to survive inside DCs comparable to those performed in macrophages [20,34,35]. In this study we use an *M. tuberculosis* genome-scale transposon mutant library, generated in the clinical W-Beijing isolate, GC1237 [36], to infect human monocyte-derived DCs and monitored mutant fitness by parallel sequencing. These data comprehensively describe, for the first time, the mycobacterial genes required for survival in the DC and so identify cellular functions and components that are critical to the interplay between the DC host cell and *M. tuberculosis* pathogen.

## Results and Discussion

### Generation of a transposon mutant library in *M. tuberculosis* GC1237

A clinical W-Beijing strain of *M. tuberculosis,* GC1237 [36], was transduced with the Mycomar phage and plated onto 7H11 to generate a library of approximately 2 × 10^5^ independent insertional mutants. This was a similar size to equivalent libraries generated previously in the laboratory strain, *M. tuberculosis* H37Rv [37]. Subsequent parallel sequencing of the transposon insertion sites and analysis with accumulation curves predicted that the library contained transposon insertion into 55,516 of the 62,488 predicted TA dinucleotides of the *M. tuberculosis* GC1237 genome (Additional file 1: Figure S1).

### The dynamics of *M. tuberculosis* infection of DCs

The ability of intracellular *M. tuberculosis* to proliferate in DCs remains poorly characterised. Some studies have described intracellular growth [38-40] in DCs, while others have reported only static bacterial loads [29,30]. To clarify these contradictory observations, and to place the results of the library selection in context, we examined the dynamics of the *M. tuberculosis* library during the DC infection.

**Figure 1:**
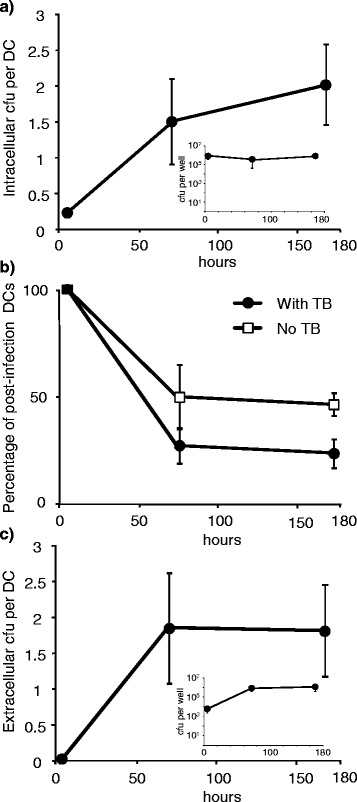
Dynamics of intracellular and extracellular *M. tuberculosis* and their dendritic cell host. The number of **(a)** intracellular *M. tuberculosis* per DC **(b)** the percentage of viable post-infection DCs and **(c)** the number of extracellular *M. tuberculosis* per DC. Total numbers of bacteria per well are shown in the plot insets. Error bars are standard errors of the mean from 5 separate experiments each with different PBMC donors.

Dendritic cells (differentiated from donor monocytes, and with surface markers characteristic of immature DCs (Additional file 2: Figure S2)) were infected with the transposon library for 4 h at a multiplicity of infection (MOI) of 5, resulting in uptake of approximately 8 × 10^5^ cfu per well (a number sufficient to minimise stochastic sampling errors), and equivalent to 23% of DCs containing a bacillus (Inset of Figure 1a). During the first 72 hours the number of DCs decreased to 27% of their initial number (44% of those in the uninfected control) (Figure 1b), behaviour similar to that observed by both Ryan *et al.* [41] and Abdalla *et al.* [42]. Total intracellular bacterial numbers in the DC cultures suggested that there was only limited bacterial growth during the infection (Inset of Figure 1a). However when intracellular *M. tuberculosis* counts were normalised to the number of live DC (Figure 1b) the number of *M. tuberculosis* per cell did increase during the first 72 h from an average of 0.24 cfu per DC to an average of 1.5 cfu per DC (Figure 1a). These bacterial loads are very similar to those that have been observed in DCs isolated from *M. tuberculosis* infected mice [4]. This rapid increase in the intracellular bacterial load was likely due, at least in part, to the re-uptake of bacilli released from lysed DCs and observed in the extracellular fraction (Figure 1c). Between day 3 and day 7 the DC population stabilised and the average bacillary load per DC did not rise significantly (although donor-to-donor variability was considerable) indicating only minimal intracellular bacterial growth (Figure 1a and Figure 1b).

### The genetic requirements of *M. tuberculosis* for DC infection

To assess, in parallel, the relative fitness of each mutant within DCs, DNA from the intracellular *M. tuberculosis* mutants was extracted 4 hours, 3 days and 7 days after infection, and the position and frequency of transposon inserts determined.

**Figure 2:**
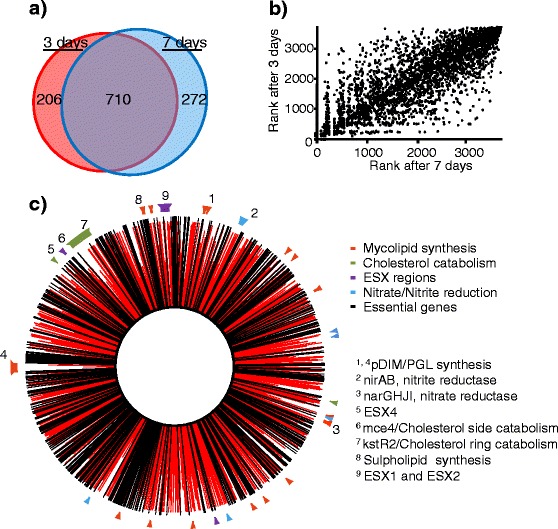
Overview of mutant ‘fitness’ during dendritic cell infection. **(a)** A Euler diagram (Venn diagram with areas proportional to the values) showing the numbers of mutants with significant fitness costs (p < 0.05) at different times during *M. tuberculosis* infection of DCs (Additional file 1), **(b)** a plot of the ‘fitness’ rank of mutations in individual genes 3 days and 7 days after infection, Spearman’s rank-order correlation ρ = 0.856, p < 1 × 10^−6^. **(c)** a genome plot of the ‘fitness’ of intracellular *M. tuberculosis* mutants 7 days after infection. Red lines indicate fitness of the gene knockouts 7 days after DC infection. Black lines are genes assigned as essential *in vitro* [43] and so excluded from the analysis. Relevant regions of co-functionality are indicated around the perimeter.

Our data identifies a large number of mutants as having fitness costs during survival in DCs (Additional file 4). Of the genes identified as non-essential *in vitro* [43], mutations in 982 genes had fitness costs after 3 days and 916 after 7 days (Figure 2a); these gene sets correlated strongly with each other (Spearman’s rank-order correlation = 0.856, p < 1 × 10^−6^, Figure 2b), indicating that much of the selection took place during the first 3 days, with only a few extra genes having fitness effects between days 3 and 7. This perhaps reflects the stability of the DC and *M. tuberculosis* populations during this time (Figure 1).

**Figure 3:**
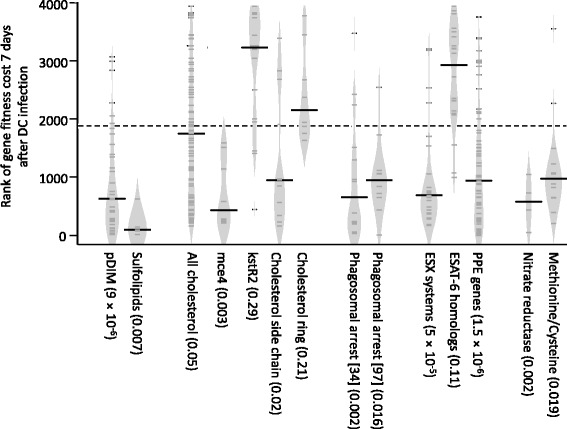
Violin plots of the ‘fitness’ of mutants ordered by functional groups 7 days after dendritic cell infection. Violin plots describing the distribution of the genes within functional gene groupings ranked by their ‘fitness’ associated p values 7 days after DC infection. Violin plots [139] are similar to box plots, but use a kernel density estimation of the probability density, similar to a smoothed distribution histogram (light grey zone), to better illustrate the overall distribution of the data. The probabilities that mutations of the genes of the functional groups had a decreased intracellular fitness, calculated using the Mann–Whitney *U* test, are given in brackets. Median values are indicated by the bold black lines, each gene by smaller dark grey lines, the dashed line represents the median rank for all non-essential genes [43] in the data-set.

These ‘fitness’ associated genes implicate a diverse range of functions and structures as being important for *M. tuberculosis* survival in DCs, many of which are known virulence factors (p = 4 × 10^−8^ as reviewed by Forrellad [18]) or have been shown to be important for *M. tuberculosis* survival in macrophages [21]. Mutants with fitness costs included multi-gene loci associated with specific functional groups such as: the synthesis of lipids *e.g.* phthiocerol dimycolates (pDIMs) [44], phenolic glycolipids (PGLs) [45] and sulfolipids; cholesterol metabolism [46-48] and ESX systems [49] (Figure 2c and Figure 3). Other functions associated with fitness are more scattered across the genome, but included PPE genes, the genes required for nitrate reduction and genes involved in the metabolism of methionine/cysteine.

### Gene-Nutrient Interactions

Gene-nutrient interaction analysis was used to interpret our genome-scale dataset [50] and to inform further *ex silico* analysis. This powerful method combines the mutant’s intra-cellular ‘fitness’ data with a metabolic model (GSMN-TB [51]) to make unbiased predictions about the importance of different metabolic pathways. This analysis predicted several bacterial nutrients/pathways to be important during DC infection (Additional file 5) such as cholesterol catabolism, propionyl and valerate degradation (which is likely to be associated with the cholesterol degradation), nitrate reduction, and methionine/cysteine metabolism, as well as several that are well established as being required for intracellular survival, *e.g*. Fe, O_2_ and CO_2_. Although powerful, this approach was somewhat constrained by the redundancy inherent in *M. tuberculosis* metabolism and by the wide variety of theoretical substrates available to *M. tuberculosisin silico* and so our interpretation of these outputs remains cautious. It is perhaps most useful when used to independently direct further *ex silico* analysis.

### Cell wall lipids are important for *M. tuberculosis* survival in DCs

The lipids of the mycobacterial cell wall are well established as important virulence factors both *in vivo* and in macrophages [18]. The present study confirms that many of these cell wall lipids are also of primary importance for fitness in DCs. Loci involved in their synthesis and modification were amongst the genomic regions most strongly associated with fitness costs.

*Mycoserate containing lipids: pDIMs and PGLs*. Mycoserates and the related lipids, phthiocerol dimycolates (pDIMs) and phenolic glycolipids (PGLs) are recognised as some of the most important *M. tuberculosis* virulence factors, particularly in Beijing strains, such as GC1237, which can have unusual pDIMs and PGLs that have been equated with hypervirulence and an ability to modulate cytokine responses [52,53].

In our DC experiments, mutations known to be associated with a loss of pDIM production (Rv2929 to Rv2942) [44] were strongly associated with fitness cost both 3 days (p = 5 × 10^−7^), and 7 days (p = 8 × 10^−12^) after the DCs were infected (Figure 3 and Additional file 4). By day 7, all the genes of the pDIM loci [44] except *drrA* had a fitness cost when mutated, as well as much of the Rv0096-Rv0101 loci, another region associated with pDIM production and virulence in mice [54]. This correlates with reports of pDIM mutants being attenuated in both mice [55] and in macrophages [56], phenotypes that have been associated with an inability to prevent phagosomal acidification [56]. Observations by Tailleux *et al.* [32] that the pDIM region was upregulated during infection of DCs, further supporting a role for pDIMs in DCs.

Mutation of many of the genes unique to the production of PGL also had a fitness cost (*pkS15/1*, Rv2957-2959c [57] and Rv2962c) at day 3 and day 7 suggesting that PGL, at least in our GC1237 strain, has a role in *M. tuberculosis* survival in DCs (Additional file 4). This is in contrast to the situation observed in macrophages infected with H37Rv, where PGL associated attenuation was only observed in pDIM-minus strains [56]. Whether this anomaly represents a difference between DC and macrophages, or whether it is associated with strain-specific difference in PGL profiles is difficult to ascertain.

*Lipids containing mycolic acids: Cord factor and sulfolipids*. Trehalose dimycolate (TDM or cord factor) and trehalose monomycolate (TMM) are another group of well characterised *M. tuberculosis* virulence factors. Cells with lowered amounts of TMM and TDM are less able to inhibit lysosomal fusion in macrophages [58] and less able to grow in macrophages [59]. In this study we observed that TDM is also an important virulence factor in DCs (Additional file 4).

As cord factor is an essential component of the mycobacterial cell wall [60] many of the genes involved in its synthesis will not be present in the library. However, some mutants that alter the amount, or change the structural details, are viable. One such group of genes is the Ag85 complex, *fbpABC,* which are all partially redundant and yet all had fitness costs in DCs (Additional file 4). These genes are involved in the transfer of mycolic acid from one TMM to another, to generate TDM and release trehalose [60]. It has been previously shown that mutating *fbpA* and *fbpC* produces *M. tuberculosis* that contains less TDM than wild type strains, that are less able to evade killing within macrophages, and that are more susceptible to reactive oxygen (ROS) and reactive nitrogen (RNI) species [61]. In other studies *fbpA* and *fbpC* have been shown to be less able to modulate the expression of MHC-II in DCs and are less able to restrict T-cell activation [60,61]. Mutants of *fbpD*, which has no TMM/TDM phenotype and is thought not to be a mycolate transferase [62], did not have fitness costs in DCs in our study.

Mutations in genes that modify the structure of the mycolic acids that are bound to the trehalose of TMM and TDM also had fitness costs in DCs (Additional file 4). The acylating genes *papA3* [63] and *pks4*, and the mycolic acid transporter, *mmpl10* [64] all had fitness costs after both 3 and 7 days in DCs. Mutantions that alter or abolish the methyl, keto and cyclopropyl modifications of these mycolic acids (*mmaA1* [65], *mmaA3* [66], *mmaA4* [67,68], *umaA* [69]) also were less fit in DCs (*mma2* and *cma2* partially complement one another [65] and did not have a fitness cost when mutated). These mutations are known to have altered virulence characteristics; *mmaA1*, *mmaA3*, *mmaA4* and *umaA* being highly attenuated and hyper-inflammatory in macrophages and in murine infection models [66,67,70].

Sulfolipids, are another group of acylated trehaloses that are abundant in the mycobacterial cell wall [71]. The ability of purified sulfolipids to modify cellular responses is well established [72,73] but there are fewer reports of a direct role in virulence. Gilmore [74] observed a decrease in intracellular growth in macrophages in sulfolipid minus mutants, while Passemer *et. al.* [56] also noted a loss of virulence in macrophages but only in pDIM minus *M. tuberculosis* strains. In our DC studies the mutations of the genes involved in sulfolipid synthesis (Rv3820-Rv3826) [75-77]] all had fitness costs (p = 0.008 and 0.007, after 3 and 7 days post-infection, respectively) as did the sulfolipid transporter, *mmpl8* [76], despite the production of pDIMs (Figure 3 and Additional file 4). Gene nutrient interaction analysis also identified cysteine/methionine metabolism as important for survival in DCs (p = 0.019 after 7 days; Figure 3 and Additional file 4). Although this may be associated with a requirement for amino acid synthesis, this seems unlikely as it is thought that methionine is available to *M. tuberculosis in vivo* [78]. However, a functional sulphur metabolism would be required for the supply of sulphur from such amino acid substrates to sulfolipid synthesis and this may in part explain the fitness costs associated with cysteine/methionine metabolism. Taken together, these data clearly show that in DCs, sulfolipids have an important and as yet, not fully elucidated, role even when pDIMs are present.

*Lipids: PIM, LM and LAM.* Phosphatidylinositol mannosides (PIM), lipomannan (LM) and lipoarabinomannans (LAM) are also essential components of the *M. tuberculosis* cell wall and so genes required for their synthesis [79,80] will not be present in the library. Both PIM and LAM have been implicated in survival in macrophages, by amongst other mechanisms, the inhibition of phagolysosomal fusion [81], and the modulation of both cytokine responses and of the phagosomal maturation process [25]. Much of this activity is thought to require LAM with an intact mannose cap [82], although cap-less mutants (∆Rv1635c) have been reported to have an unaltered wild-type phenotype in murine macrophages and *in vivo* [83]. In our human DC experiments, mutants predicted to result in a non-mannose capped LAM (Rv2181, Rv1635c, Rv1565c) all had a fitness cost both 3 days and 7 days post-infection (Additional file 4). This is consistent with studies showing that *M. tuberculosis* binding to human DCs involves interaction of manLAM with the receptor DC-SIGN [29,84].

### Cholesterol side chain catabolism contributes to fitness in DCs

Cholesterol is both an important nutrient for intracellular *M. tuberculosis* and a critical part of the host cell’s response to *M. tuberculosis* [85]. As such, the catabolism of cholesterol has multiple functions during macrophage infection. It provides propionyl-coA precursors for the synthesis of pDIM [86,87] and the sulfolipid, SL-1 [88] via methyl malonyl-coA. It can be used to supply carbon to central metabolism of *M. tuberculosis* as succinate and pyruvate [86]. Finally, the degradation of host cholesterol can be involved in the modulation of intracellular trafficking and immune signalling [89-91]. Prior to this present study, it was not known which of these functions, if any, were important during DC infection, although cholesterol catabolism genes and the associated *kstR* regulon [92] were known to be upregulated [32]. Our data provides evidence for the importance of cholesterol catabolism during DC infection (p = 0.05, Figure 2c and Figure 3), an interpretation confirmed by the gene-nutrient interaction analysis (Additional file 5). However fitness costs were mainly associated with mutants of the *mce4* cholesterol transporting operon [47](p = 0.0026 after 7 days), genes involved in early steps in cholesterol catabolism eg *choD*, Rv1106c, *kstD*, and genes involved in side chain degradation [48,93] (Figure 3, Additional file 4, Biocyc pathway p = 0.0002 after 7 days), notably the igr region [94,95]. Fitness costs were not apparent for mutations in genes involved in sterol ring degrading pathways, such as *hsaA-G* (although several of these are known to have only minimal effects on growth in cholesterol e.g. *hsaEFG* [48], and the genes of the *kstR2* regulon [46,96].

The fitness costs associated with disruption of cholesterol side chain degradation pathways and the importance of mycolic acid synthesis during DC infection together suggests that a critical function of cholesterol degradation is to supply propionyl-CoA for the synthesis of pDIM and SL-1. Both which are virulence factors that we have shown are important for survival in DCs. However, an absolute requirement for cholesterol derived carbon seems unlikely, as the genes required for the release of carbon from the sterol component of cholesterol [48] did not have fitness costs. There is however evidence for a role for cholesterol catabolism in modulating the DC response to *M. tuberculosis*. Consistent with previous findings in macrophages, our data indicates a fitness cost for mutations in c*hoD*. ChoD is an extracellular cholesterol oxidase that when mutated does not abolish the *M. tuberculosis* ability to grow on cholesterol, but does attenuate *M. tuberculosis* in macrophages via inhibition of TLR2 signalling [91] and reduction of iNOS and ROS responses [89].

### Genes that prevent phagosomal maturation in macrophages also have a fitness cost in DCs

*M. tuberculosis* residing in both DCs and macrophages are contained within phagosomes, whose development has been arrested at an early stage in the endocytic pathway [29]. However macrophage and DC phagosomes do differ in other respects: DC mycobacterium-containing phagosomes undergo only limited acidification and have more restricted connectivity to other parts of the endocytic system [29,31]. Although we do not assay for a phagosome maturation phenotype in this study, previous studies have demonstrated that in general *M. tuberculosis* mutants with an abrogated ability to inhibit macrophage phagosomal maturation are also attenuated for intracellular survival [34]. We compared our intra-DC fitness data with published data-sets generated using genome-wide screens for mutants that are defective in their ability to inhibit murine macrophage phagosome maturation [34,97,98]. We observed that many of these phagosome-control mutants also had reduced fitness in DCs at both 3 and 7 days post-infection (p = 0.058 /0.0027 [34,97] and p = 0.016/0.0023 [34,97] respectively) (Figure 3 and Additional file 4). Similarly, many genes recognised individually as involved in modulating phagosomal maturation *e.g. sapM* [99], PE_PGRS62 [100], PE_PGRS30 [101], *eis* [102] also had fitness costs. Finally, many of the virulence associated functions and structures such as cell-wall lipids and LAM [103], that we have already identified as important for fitness in these DCs are known to be involved in preventing phagosomal maturation, properties which may in part explain the intra-DC fitness costs of mutating these genes. We conclude that despite the difference between DC and macrophage phagosomes, control of phagosome-lysosome fusion remains as important for *M. tuberculosis* fitness in human DCs as it is in macrophages and is modulated using similar mechanisms.

### A novel role for ESX transport systems in DC

There are five ESX/type VII secretion systems/regions in the *M. tuberculosis* genome, of which two are essential for *in vitro* growth on microbiological medium (ESX-3 and ESX-5) [104,105]. We show that mutations, particularly in the pore-forming components [49], of the other three ESX systems (ESX-1, ESX-2 and ESX-4) all had fitness costs for growth in human DCs (p = 0.0007, 0.0005 and 0.042 respectively after 7 days, Figure 3 and Additional file 4). ESX-1 is the best characterised of these systems and is involved in the export of the classic virulence factors, ESAT-6, CFP-10 and many Esp proteins. ESX-2 and ESX-4 are less well studied and until now have not been recognised as virulence determinants [106].

Unexpectedly, the intra-DC fitness cost associated with loss of the ESX-1 secretion apparatus were not found to be associated with mutants of the canonical ESX-1 substrates, ESAT-6 and CFP10 (Figure 3 and Additional file 4). This discrepancy, which has been observed previously in mice [107,108], implies an additional function for the ESX-1 system that is important for survival and virulence in DCs. Work by Joshi *et al.* [109] with an *eccA1* mutant of *M. marinum* supports such a conclusion, with evidence that ESX-1 is not only required for the export of ESAT-6 type proteins, but also for the export of enzymes involved in the synthesis of mycolic acids, pDIMs and PGLs *e.g.* Pks13, KasB, KasA, MmaA4, Pks5, Mas, Pks15/1, PpsD, and PpsE. This links with our findings that production of wild-type amounts of these lipids is important for survival in DCs. Many PPE genes are also thought to be exported by ESX systems [49,110]. Indeed, in *M. marinum* ESX-5 has been shown to export many of the PPE proteins [111,112]. These PPE genes are defined by a proline-glutamate-glutamate motif, but may have varying roles in *M. tuberculosis* physiology and virulence and remain poorly characterized (for a review see Akhter *et al.*, 2012 [113]). This study shows strikingly that many of these PPE proteins are important for fitness in the human DC with over half of the annotated PPE mutants lost from the library by day 7 (p = 1.5 × 10^−6^), including many of the PPE genes chromosomally and functionally associated with ESX-5 in *M. marinum* (PPE28, PPE29, PPE30, PPE31, PPE32 and PPE33) [111]. It seems reasonable to extrapolate that in *M. tuberculosis*, ESX-5 may also secrete these PPE proteins and potentially many of the numerous other PPE proteins that we have shown are important for intra-DC virulence. Thus we believe ESX-5 is an important virulence factor in *M. tuberculosis*.

### *M. tuberculosis* responses to oxidative and nitrosative stress

Human DCs are thought to produce less ROS than macrophages in response to *M.* tuberculosis infection [32,114] and are more resistant to ROS associated apoptosis. *M. tuberculosis* resistance to ROS is mediated via a range of genes and cellular components, such as the cell wall, which scavenges free radicals, and protein and DNA repair systems, which are actively involved in withstanding ROS stress [115]. Many genes associated with ROS resistance do have fitness costs in DCs (Additional file 4): genes of methionine/cysteine metabolism (as identified by gene-nutrient interaction analysis) [116]; glutamyl-cysteine ligase; Rv1806 to Rv1809 (PPE20, PPE30-33) [117]; as well as many genes involved in the synthesis of the cell wall components such as LAM, PGL and mycoseric acid. However it is difficult to distinguish a ROS specific role for many of these pleiotropic genes. Specific evidence for significant ROS associated fitness costs in DCs is also difficult to equate with the lack of fitness cost for genes such as those of the oxidized guanine (GO) DNA repair system [118], *lsr2* [88], *ahpC* [119], or mycothiol pathways [120,121], making predictions as to the role of ROS in *M. tuberculosis* infection of DCs unclear, but suggesting that it does not represent a dominant antimicrobial mechanism.

The role of nitrogen radicals in human DCs is also not well established. Murine DCs are known to produce a nitrosative burst upon *M. tuberculosis* infection, but there is less evidence for an equivalent response in humans [32,122]. We find that many of the genes that have been associated with resistance to reactive nitrogen in macrophages are also important during DC infection *e.g. fbiC*, Rv2115c, Rv2097 [123], the nucleotide excision repair system encoded by *uvrABCD1D2* [124] and nitrate reductase associated genes (*narG*, narH, *narI*, *narK1*, *narK2*, *narX*, *nirB*, p = 0.0021, Figure 3 and Additional file 4). In macrophages, nitrate reductase is important for many of the *M. tuberculosis* responses to reactive nitrogen. An active nitrate reductase in the presence of NO, has been shown to promote *M. tuberculosis* growth [125], to be a source of nitrite (which could then acts as signal molecule for *M. tuberculosis*) [122] or potentially disrupt NO signalled apoptosis [126]. Without nitrate reductase *M. tuberculosis* is more susceptible to both acid [127] and peroxide stress [128]. Our data imply that NO, or at least nitrate, is indeed present in the phago-lysosome of human DCs. This requirement for nitrate reductase is unlikely to be associated with resistance to acid (at least in DCs) as has been described for macrophages [127] but more likely relates to its other growth promoting roles such as providing an alternative electron acceptor, making available a source of metabolic nitrogen, or as a mechanism of preventing apoptosis.

## Conclusions

The ability to survive within DCs is integral to the ability of *M. tuberculosis* to manipulate and on occasions usurp the host's immune response. As such the genetic requirements for these processes represent important *M. tuberculosis* virulence components. By using high-throughput analysis of transposon libraries we have produced a comprehensive genome-scale assessment of the genetic requirements of a clinical *M. tuberculosis* W-Beijing isolate, GC1237, for survival in DCs. Such an approach connects gene and function, allowing analysis at multiple scales: from single loci, regulon, metabolic pathway, and even whole cell, rather than on the gene-by-gene basis produced by more conventional gene knockout strategies involving one or two mutants. Such a strategy has been used previously with the laboratory strain *M. tuberculosis* H37Rv, to successfully describe the genes required for a range of virulence related environments: *in vitro* growth [48]; infection of mice [37]; survival in murine macrophage, human cell lines and murine bone marrow derived macrophages [21]; and for the ability to inhibit phagosome maturation [22,34,97,98].

**Figure 4:**
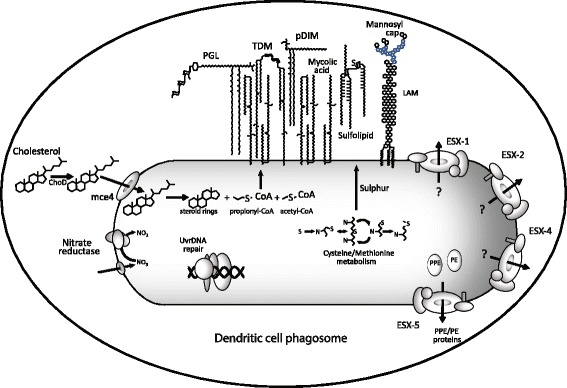
Summary of the major cellular components and processes that are associated with fitness costs during *M. tuberculosis* survival within DC.

Our data shows that the survival of *M. tuberculosis* within human DCs requires a large number of genes (see Figure 4 for a summary), many of which have previously been recognised as also being important during the invasion and survival of macrophages. Perhaps the best characterised of these are the genes involved in the synthesis, transport and modification of cell wall lipids. These lipids are clearly very important to the ability of *M. tuberculosis* to survive within DCs. Our data demonstrates that not only are the well-established virulence associated lipids important such as pDIM and cord factor, but also lipids that had previously only been ascribed secondary roles such as PGL and sulfolipids [56]. These secondary lipids have long been recognised as modulators of macrophages when added exogenously [74], or associated with modulating cytokine production [52,53] and virulence in mice, but have not previously been shown to have a direct effect on intracellular virulence of *M. tuberculosis* [56] and may be especially important during DC infection. The apparent requirement for *M. tuberculosis* GC1237 to have functional cholesterol catabolism and sulphur metabolism when in DCs may also be linked to the supply of the metabolites required for the synthesis of these cell wall lipids, although roles in nutrition or immune modulation cannot be ruled out.

In macrophages, cell wall lipids act, in part, by modulating the phagosomal environment. Despite differences in the physiology and cell biology of macrophage and DC, mycobacterial modulation of the phagosome remains important in DCs, with similarities between genes required for survival in DCs and groups of genes identified as required for phagosomal modulation in macrophages. The ESX-1 secretion system is well established as a fundamental virulence mechanism of *M. tuberculosis*, being involved in host-pathogen interactions including phagosome escape and cytotoxicity. Here, we demonstrate a role during DC infection for not only ESX-1 but also ESX-2 and ESX-4, and a requirement for PPE genes, which implicates ESX-5 as also having a direct role in mycobacterial pathogenesis. The functions of the abundant and characteristic PPE gene/protein family have remained elusive but we show, for the first time, that the intra-DC environment represents a powerful non-redundant selection for their maintenance in the genome. We also identify important roles for sulphur and nitrogen metabolism. Cysteine/methionine metabolism is an important fitness determinant, possibly related to resistance to reactive species or to the production of sulfolipids. Nitrate reductase was also important during survival within DCs, and again the mechanism is unclear, but resistance to reactive nitrogen intermediates, nitrogen acquisition, or a role in intercellular signalling would seem likely candidates.

Clearly, many *M. tuberculosis* intracellular virulence mechanisms are common to both macrophages and DCs. This may reflect the close relationship between the myeloid DC and the macrophage which represent different but related cell types within a single mononuclear phagocytic system. However, we also identify novel DC virulence mechanisms that are important in human DC infection but which are either absent or have been overlooked in macrophages. Further investigations into the mechanisms that underlie the importance of these functions during infection are required to further our understanding of the nature of the inter-relationship between host and pathogen during pathogenesis.

## Methods

### *M. tuberculosis* culture and library construction

*M. tuberculosis* GC1237, a W-Beijing strain [36] was routinely grown at 37°C on Middlebrook 7H11 plates containing 10% OADC and 0.2% glycerol or in 7H9 media containing 10% ADC, 0.2% glycerol and 0.05% Tween 80®. *M. tuberculosis* were enumerated by plating 10 μl drops of serially diluted culture onto 7H11 plates. *M. smegmatis* mc^2^ were grown in Nutrient Broth II with 0.2% glycerol and 0.05% Tween 80® or on solid Nutrient Broth II media with 0.2% glycerol. The temperature sensitive phage, phAE87 [129] containing pMycoMar [130] was prepared by adding an aliquot of late log phase *M. smegmatis* to Nutrient Broth II top agar with an appropriate number of phage such that the resultant plaques were touching. After 3 days at 30°C the top agar was scraped from the plates and shaken with an equal volume of MP buffer (150 mM NaCl, 50 mM Tris, 10 mM MgSO_4_, 2 mM CaCl_2_, pH 7.4), the preparation was centrifuged, the supernatant passed through a 0.22 μM filter, and the phage enumerated. The *M. tuberculosis* library was prepared by washing an *M. tuberculosis* GC1237 culture, of approximate OD600 1.0 with an equal volume of MP buffer and resuspending in 0.1 volumes of phAE87 at an multiplicity of infection (MOI) of between 10–100, incubating at 37°C for 4 h and plating on 7H11 media containing kanamycin at 30 μg ml^−1^. The resultant library was scraped from the plates and stored at −80°C in 10% glycerol.

### Generation of monocyte derived dendritic cells

Monocytes were isolated from leukocyte cones (National Health Service Blood and Transplant, UK), from 5 different donors. For each, the cones were diluted with PBS containing 2 mM EDTA, layered onto HistoPacque®-1077, centrifuged at 400 × g for 30 min and allowed to come to rest without braking. Monocytes were purified from the resulting buffy coat using CD14 Microbeads (Miltenyi, Bisley, UK) as recommended by the manufacturers. Approximately 1 x 10^7^ monocytes were differentiated into DCs in 75 cm^2^ flasks incubated at 37°C with 5% CO_2_. DCs were grown in RPMI 1640 containing GlutaMax™ (Invitrogen, Paisley, UK), supplemented with 10% Fetal Calf Serum (FCS) and 10 ng ml^−1^ GM-CSF (PeproTech, London, UK) and 20 ng ml^−1^ IL-4 (PeproTech). After 6 days the differentiated cells were washed three times in PBS by centrifugation, resuspended in fresh growth media and 3 × 10^6^ cells per well cultured in 6 well tissue culture plates.

### Flow Cytometry

DCs were washed in FACS buffer (PBS containing 5 mM EDTA, 1% FCS, 0.05% sodium azide). The cells were resuspended in FITC or PE conjugated antibody for CD14, CD83, CD86, CD1a or appropriate isotype controls diluted 1:100 or 1:20 with FACS buffer. The cells were incubated on ice for 30 min and then washed twice in FACS buffer, before resuspending in 4% paraformaldehyde in PBS. Samples were run on FACS Canto™ (BD Biosciences, Oxford, UK) recording 10,000 events.

### Infection of dendritic cells with *M. tuberculosis*

Each DC preparation was infected with a previously frozen aliquot of the *M. tuberculosis* library cultured for 24 h in 7H9 with ADC but without Tween 80®. Broths were centrifuged and resuspended in DC growth media, and passed multiple times through a 26G needle to disperse clumps. The inocula concentration was estimated by measuring optical density at 600 nM and diluted in DC growth media to give a MOI of 5. The *M. tuberculosis* were added to the DCs and the infection incubated at 37°C for 4 h. Control wells contained either no *M. tuberculosis* or no DCs.

After the infection, *M. tuberculosis* that were not internalized were removed by washing the wells three times with DC growth medium. Planktonic DCs in these first washes were then separated from the bacteria by combining the supernatants, and centrifuging at 500 × g for 5 min three times, each time removing the supernatant and resuspending the cell pellet in fresh DC growth medium. The supernatants were combined and the “extracellular” *M. tuberculosis* recovered by centrifuging at 3500 × g for 5 min and enumerated by plating on 7H11 and counting colony forming units.

The intracellular fraction was prepared by resuspending the planktonic cell pellet from the extracellular preparation in 0.1% Triton X-100® and adding this back to the adherent cells remaining in the well. This lysate was centrifuged at 3500 × g for 5 min, the bacterial pellet resuspended in an appropriate volume, enumerated and the remainder spread on plates to recover the library for DNA purification and TnSeq analysis (see below). For DC counts, the growth media was removed from a well and centrifuged at 500 × g to give a cellular pellet. Versene™ (Invitrogen) was added to the well and the plate incubated at 37°C for 10 min. The Versene™ was removed and used to resuspend the cellular pellet, and live and dead cells counted using a Trypan Blue exclusion assay. Infections were sampled immediately after washing (4 hours), after 3 days and after 7 days. Recovered libraries were scraped from the plates and stored at −80°C in 10% glycerol.

### DNA purification and preparation

DNA was extracted from recovered libraries from three of the infections. An aliquot of each recovered library was added to a lysing matrix B tube (MPBiomedicals, Loughborough, UK) containing 25:24:1 phenol:chloroform:isoamyl alchohol and TE, and mechanically disrupted in a Fastprep™ FP120 (MPBiomedicals) at setting 4 for 30 sec. The preparation was centrifuged at 12,000 × g for 5 min, and the supernatant washed twice with chloroform, and precipitated with × 0.7 volumes isopropanol and × 0.1 volumes 3 M sodium acetate. DNA was resuspended in water and sheared by sonicating 3 times for 30 sec each (1 sec on/1 sec off) with a VibraCell® VC300 and a 6 mm probe. Sheared DNA was repaired by incubating with 100 U μl^−1^ T4 DNA polymerase (Promega, Southampton, UK), 50 mU μl^−1^ Klenow (Promega), 100 mU μl^−1^ polynucleotide kinase (New England Biolabs, Hitchin, UK), 2 mM dNTPs all in × 1 T4 DNA ligase buffer (Promega) for 30 min at 20°C. Repaired DNA was purified using QIAquick™ columns (Qiagen, Crawley, UK), and end labelled with A nucleotides by incubating with 0.25 U μl^−1^ Klenow, 0.2 mM dATP in × 1 Klenow buffer (Promega) for 30 min at 37°C and purified on MiniElute™ PCR purification kits.

### Transposon amplification

A linker was generated by annealing oligonucleotides linker1 and linker2 (Additional file 3: Table S2a) at equimolar concentrations in 50 mM NaCl, 5 mM Tris, 0.5 mM EDTA, pH 8 by heating the mix to 95°C for 2 min and then cooling for approximately 1 h to 20°C. The linker was annealed to the sheared genomic DNA at eqimolar ratios of fragment ends using T4 DNA ligase incubated at 16°C for 18 h. The fragments were PCR amplified with 0.2 μM PCR-linker primer and either 0.2 μM primer PCR-MarinerA or PCR-MarinerB (Additional file 3: Table S1), using 0.02 U μl^−1^ KOD Hot Start DNA polymerase (Novagen, Feltham, UK), ×1 KOD buffer, 0.2 mM dNTPs and 1.5 mM MgCl_2_. Conditions were 94°C for 2 min, followed by 5 cycles of 95°C for 30 sec, 69°C for 10 sec, 70°C for 30 sec, followed by another 5 cycles with an annealing temperature of 67°C, followed by 20 cycles with an annealing temperature of 65°C. Products sized between 250–500 bp were cut from an agarose gel, cleaned with QIAquick columns, and equal amounts of each PCR product (MarinerA and MarinerB) combined and amplified with primers PCR2-linker and PCR2-Mariner as before but with cycle conditions of 94°C for 2 min, followed by 9 cycles of 95°C for 30 sec, 57°C for 10 sec and 70°C for 30 sec. Fragment sizes were assessed and products quantified with a BioAnalyzer 2100 (Agilent). The indexed products from different samples were combined, spiked with 50% PhiX and sequenced on an Illumina® 2000 HiSeq™ machine, with a single index and a single read of 100 cycles.

### Sequence analysis and statistical analysis

Sequence reads were assigned to samples according to their indices and aligned to the GC1237 genome using Bowtie2 [131]. Transposon TA junctions were identified from sequence reads that contained a fragment of GC1237 genome preceded by the terminal-end of the transposon sequence (5’ CGGGGACTTATCAGCCAACCTGT, 2 mismatches were permitted). Mutant fitness was determined by comparing transposon frequencies between time points (between 4 h and 3 days, and 4 h and 7 days) using a method based upon the logistic distribution of the difference of two extreme value random variables [132]. This approach depends upon the difference in length of the two longest transposon-free runs at two time points with null hypothesis of zero difference. Values for the three repeats were combined using Fisher’s combined probability test as reviewed by Loughin, 2004 [133], and a value of p < 0.05 was considered to represent a significant loss of fitness. Correlations between the library at different time points were assessed using the Spearman’s rank order coefficient. The significance of gene group enrichments was determined using the Mann–Whitney *U* test (Wilcoxon rank-sum test).

### Accumulation curves

To determine the library coverage the number of unique TA sites mutated was plotted against the number of insertions sequenced. Asymptotes were calculated by fitting Michaelis-Menten curves to the plots.

### Gene Nutrient Interactions

Gene-nutrient interaction (GNI) analysis, as developed by Idit *et al.* [50], aims to describe the relationship between gene essentiality and the presence or absence of nutrients in the growth environment. Using the approach, the 101 nutrients from the GSMN-TB *M. tuberculosis* metabolic model [51] were randomly sampled to generate 1.17 × 10^6^*in silico* growth media. Flux balance analysis was carried for each growth media to identify essential genes. A gene was considered essential if the drop in the growth rate after its knockout was more than 20 %. Positive/negative GNI were identified when a nutrient was present/absent in a significantly number of media under which the gene was essential, as estimated by calculating a p-value based on the hypergeometric distribution. The calculation above has been implemented as an extension to Surrey FBA software [134] and is available on request.

### Availability of supporting data

All raw sequence read files have been submitted to the Sequence Read Archive of the National Center for Biotechnology Information under SRA Study accession, SRP057496.

## Additional files

Additional file 1: Figure S1. An accumulation curve of the transposon insertion sites in the *M. tuberculosis* library. Additional file 2: Figure S2. Flow cytometry data of surface markers of differentiated PBMCs. Additional file 3: Table S1. A list of primers used in this study. Additional file 4:
**An Excel file containing p values associated with the fitness during survival in DC, arranged by functional group.**
Additional file 5:
**An Excel file containing the results of gene nutrient-interaction analysis.**

## Abbreviations

DC: Dendritic cells
GNI: Gene nutrient interaction
LAM: Lipoarabinomannans
LM: Lipomannan
MOI: Multiplicity of infection
Mtb: *Mycobacterium tuberculosis*
PBMC: Peripheral blood monocytes
PBS: Phosphate buffered saline
PCR: Polymerase chain reaction
pDIM: Phthiocerol dimycolates
PGL: Phenolic glycolipids
PIM: Phosphatidylinositol mannosides
ROS: Reactive oxygen species
TCA: Tricarboxylic acid cycle
TDM: Trehalose dimycolate
TMM: Trehalose monomycolate
