# Supplementary material for: Lipid metabolism and Type VII secretion systems dominate the genome scale virulence profile of Mycobacterium tuberculosis in human dendritic cells
Source: BMC Genomics. 2015 May 9;16(1):372. doi: 10.1186/s12864-015-1569-2 (PMC4425887; doi:10.1186/s12864-015-1569-2)
Supplement: Additional file 1: Figure S1. — An accumulation curve of the transposon insertion sites in the M. tuberculosis library. [file 12864_2015_1569_MOESM1_ESM.pdf]

Additional Figure 1

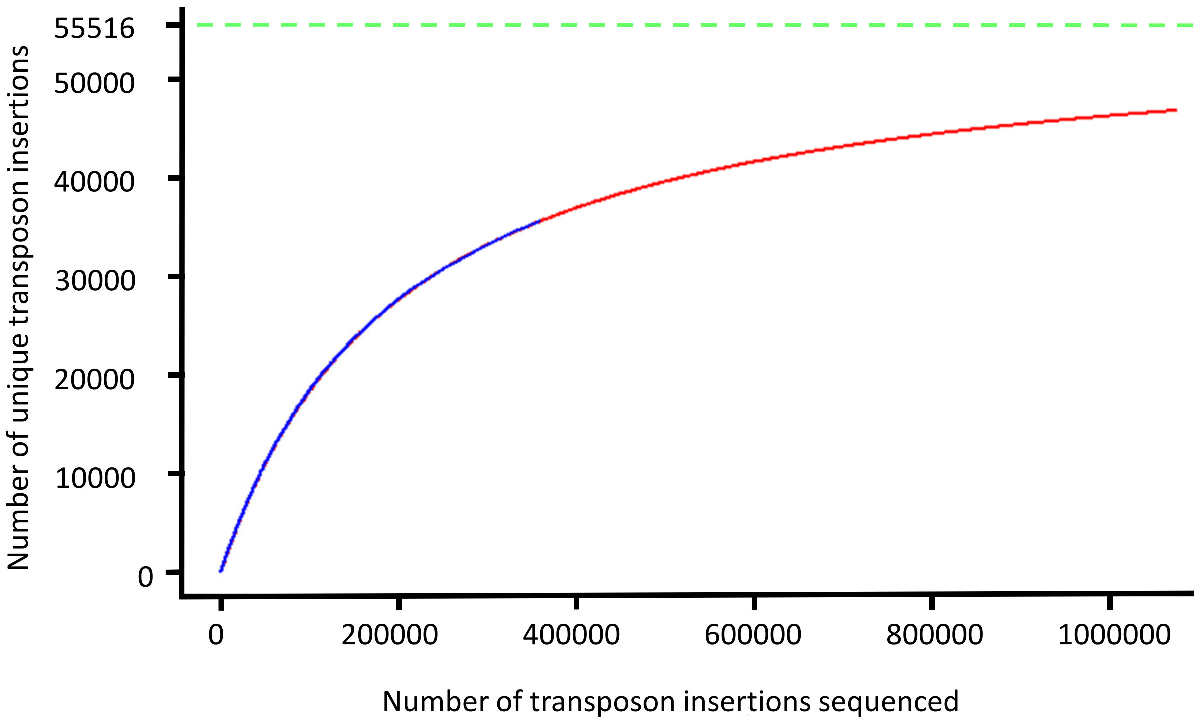

Additional Fig. 1. An accumulation curve of the transposon insertion sites in the *M. tuberculosis* library. To determine the library coverage the number of genes or TA sites mutated was plotted against the number of insertions sampled. A Michaelis-Menten curve was fitted to the data (blue line) and extrapolating (red line) to give an asymptotes (green dashed line) that predicts that the library contains 55,176 unique transposons mutants (out of a possible 62,418 predicted TAs in the GC1237 genome).
