# Supplementary material for: Lipid metabolism and Type VII secretion systems dominate the genome scale virulence profile of Mycobacterium tuberculosis in human dendritic cells
Source: BMC Genomics. 2015 May 9;16(1):372. doi: 10.1186/s12864-015-1569-2 (PMC4425887; doi:10.1186/s12864-015-1569-2)
Supplement: Additional file 2: Figure S2. — Flow cytometry data of surface markers of differentiated PBMCs. [file 12864_2015_1569_MOESM2_ESM.pdf]

Additional Figure 2

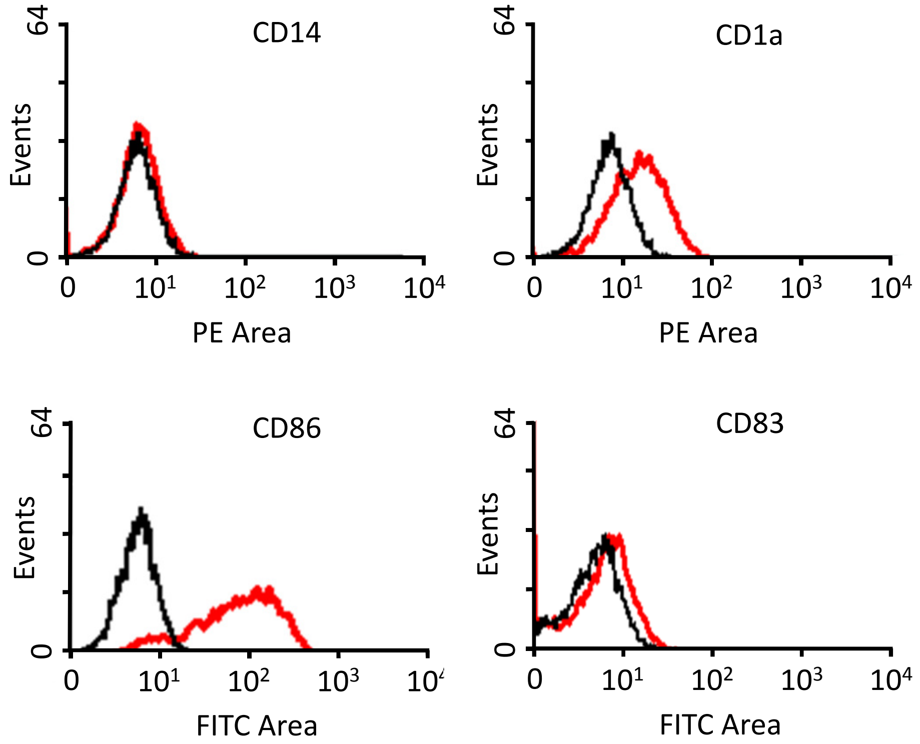

Additional Fig. 2. Flow cytometry data of surface markers of differentiated PBMCs. DCs from donor PBMC were differentiated with GM-CSF and IL-4. Flow cytometry showed that these cells had acquired a phenotype characteristic of immature DCs, a) losing their monocyte associated CD14 marker and gaining the DC markers b) CD1a and c) CD86. Upregulation of the DC maturation marker d) CD83, was minimal.
