# Supplementary material for: Lipid metabolism and Type VII secretion systems dominate the genome scale virulence profile of Mycobacterium tuberculosis in human dendritic cells
Source: BMC Genomics. 2015 May 9;16(1):372. doi: 10.1186/s12864-015-1569-2 (PMC4425887; doi:10.1186/s12864-015-1569-2)
Supplement: Additional file 3: Table S1. — A list of primers used in this study. [file 12864_2015_1569_MOESM3_ESM.docx]

**Additional Table 1a**

Oligonucleotides used to prepare, amplify and sequence Mariner transposon junctions.

| **Name** | **Sequence** |
| --- | --- |
| Linker 1^*^ | ACCACGACCA -PO_4_ |
| Linker 2 | AGTCTCGCAGATGATAAGGTGGTCGTGGTT |
| PCR1-MarinerA | CCCGAAAAGTGCCACCTAAATTGTAAGCG |
| PCR1-MarinerB | CGCTTCCTCGTGCTTTACGGTATCG |
| PCR1-linker | GTCCAGTCTCGCAGATGATAAGG |
| PCR2-linker | CAAGCAGAAGACGGCATACGAGATGTGACTGGAGTTCAGACGTGTGCTCTTCCGATCTGTCCAGTCTCGCAGATGATAAGG |
| PCR2-Mariner^†^ | AATGATACGGCGACCACCGAGATCTACACTCTTTCCCTACACGACGCTCTTCCGATCTNNXXXXXXCGGGGACTTATCAGCCAACC |

^*^The PO_4_ moiety prevents polymerase extension of the linker. ^†^ The double NN aid sequence resolution and the XXXXXX represent the variable indices

**Additional Table 1b**

Indices used to multiplex sequenced samples.

| **Sample** | **Index** |
| --- | --- |
| Time 5h | CGATGT |
| Time 3days internal | TTAGGC |
| Time 7 days internal | ACAGTG |
